# Duty to Warn in the Emergency Department: Three Medical Legal Cases That Illustrate Providers’ Broad Risk and Liability

**DOI:** 10.5811/cpcem.2020.5.47222

**Published:** 2020-07-20

**Authors:** Rosemary Pfaff, Ross P. Berkeley, Gregory Moore, Melanie Heniff

**Affiliations:** * Baylor University School of Medicine, Department of Emergency Medicine, Houston, Texas; † University of Nevada, Las Vegas School of Medicine, Department of Emergency Medicine, Las Vegas, Nevada; ‡ Mayo Clinic, Department of Emergency Medicine, Rochester, Minnesota; § Indiana University School of Medicine, Department of Emergency Medicine, Indianapolis, Indiana

**Keywords:** duty to warn, emergency providers

## Abstract

This article presents three medical-legal cases that define a physician’s duty to warn and include caveats on medical practice within the scope of the law. Some physicians may not recognize that these legal and liability requirements extend not only to physical danger, but also to infectious diseases, medical illness, and drug effects.

## INTRODUCTION

Many emergency physicians and providers are aware of their duty to warn in situations where a patient expresses ideation of harming another person(s) physically. However, fewer may understand the specific legal obligations of this duty and who should be warned. Also, physicians may not be aware that this legal duty extends to infectious disease, other diseases, and medications, which opens them to broad legal liability. Three cases below will give representative examples of this “duty to warn” and will be followed by other enlightening and classic cases with legal and medical caveats.

## CASES

### Case 1: Anonymous versus Anonymous – North Carolina

A patient presented to the ED on two occasions reporting thoughts of killing his wife. He seemed relatively reasonable and stated that he could control these urges and would seek psychiatric follow-up care. After discharge from the ED, he killed his wife and children. He lived in his house with the dead bodies for several weeks before killing himself. The case settled for $11.5 million.^1^

### Case 2: Washington versus Pediatric Cool Care – Washington

A 14-year-old female presented to a pediatric urgent care with symptoms of depression. She was evaluated and prescribed citalopram. After the initial visit, the patient had one follow-up appointment with a nurse practitioner in the same facility. Five months later, the patient committed suicide by overdosing on the citalopram that was prescribed. Citalopram has a black box warning advising that it should not be prescribed to adolescents as it may cause suicidal ideation. Her parents brought suit claiming that there was no discussion with the patient or her mother with regard to side effects. They also were not advised to read the package insert. The mother was not encouraged to observe her child closely for worsening symptoms or suicidal ideation. The plaintiffs also claimed that referral to a psychiatrist or psychologist for evaluation was not initiated by the primary providers either. After hours of deliberation, a jury awarded the plaintiffs $7.65 million.^2^

### Case 3: Kochik versus Hanna et al

A patient was diagnosed with partially controlled and unpredictable seizures and received treatment from onset forward. Defendant Dr. Moore, her family practice physician, and defendant Dr. Zind, a neurologist, provided the patient’s care together. Evidence of whether the physicians advised her that it was unsafe for her to drive was conflicting. Six years after the diagnosis, the patient was driving home and had a seizure, which caused her to lose consciousness and control of her vehicle. She crossed the centerline and struck an automobile carrying four people, causing their deaths. The plaintiff brought this action against the defendants for their negligence regarding their failure to warn her not to drive due to her seizure disorder. The Court found that it is clearly foreseeable that the defendants’ alleged failure to warn the patient not to drive would endanger the motoring public, which would include the decedents in this case. Specifically, the Court found that the likelihood of injury to a third party due to an automobile accident arising from the physicians’ failure to inform her not to operate a motor vehicle is not so rare or unusual an occurrence as to be considered unforeseeable. Furthermore, warning the patient that it was unsafe for others if she drove did not violate physician-patient confidentiality as those in danger would not be aware of her condition.^3^ The case has yet to be fully adjudicated for damages.

#### Ms. Pfaff

On October 27, 1969, Prosenjit Poddar killed Tatiana Tarasoff. Prior to the murder, Poddar disclosed his intention to kill Tsrasoff to his psychologist, Dr. Lawrence Moore. Dr. Moore attempted to have Poddar detained after the admission. Poddar was released after the police determined Poddar to be of a rational state of mind. Dr. Moore’s superior directed that no further action be taken in the attempt to detain Poddar. Following this sequence of events, Poddar murdered Tatiana Tarasoff by shooting her with a pellet gun and repeatedly stabbing her with a kitchen knife. Upon conviction, Poddar was diagnosed with paranoid schizophrenia, a diagnosis previously suggested by Dr. Moore during his psychiatric care.

The victim’s plaintiff parents filed a claim that the psychiatrists in question breached their duties to provide reasonable care. Initially, the claim was dismissed by the Superior Court of Alameda (California), under the assertion that Dr. Moore’s duty to provide reasonable care to Poddar, his patient, was fulfilled in his attempts to treat and detain, maintaining doctor-patient confidentiality. The plaintiffs amended their claim, citing that the psychiatrist had a duty to warn either Tatiana or her immediate family of the imminent danger. The Supreme Court of California held that the defendants did, in fact, fail in their duty to warn, weighing the societal benefits over the need to maintain patient confidentiality. This set a new precedent for the responsibilities of mental health providers.

Defendants argued that setting a precedent for a duty to warn would lead to a majority of erroneous warnings, compromising a patient’s trust in confidentiality and hindering adequate patient care. The burden of correctly identifying potentially dangerous patients would reside on the provider, and the provider would inevitably err on the side of caution and report his or her patient and warn potential victims. The court’s ruling emphasizes that these risks to the patient-provider relationship are justified by the societal good and safety that comes with the warning of a potential threat. Providers today are responsible for warning persons directly threatened by a patient, or individuals close to the potential victim, if the provider suspects a legitimate danger. As it stands, however, the law supports that a physician’s duty extends beyond that of his patient’s care, and thus a duty to warn potentially vulnerable individuals is necessary.

It is important to highlight that while many providers are aware of this landmark case, few realize that notifying the police did not absolve them of responsibility or liability.^4^

#### Dr. Heniff

Identifying those who are legally at risk is subject to vague and wide interpretation by courts, making the task very difficult for front-line healthcare providers. A recent court case further affirmed and seemed to expand the duty to warn.^5^ A patient with bipolar disorder saw a psychiatrist on an outpatient basis over the course of 10 years. The patient had a history of poor compliance with medication and on several occasions expressed homicidal and suicidal thoughts. After his wife divorced him, he suffered worsening depression and again expressed suicidal and homicidal thoughts but assured his physician that he would not act on the thoughts. Two years later, the patient fell in love and became engaged to a woman who had three sons. The woman moved herself and her sons out of the home after he hit one of her sons. The patient then saw his psychiatrist for what would be the final time and stated he was experiencing some suicidal ideation but would not act on it. He indicated that he was stable and getting back together with his fiancée and didn’t express homicidal ideation.

Three months later, the patient shot and killed his fiancée and one of her sons. He then returned to his home where he committed suicide.

The family of the victims brought suit against the psychiatrist and the psychiatric clinic. The case was appealed to the Supreme Court of Washington, which found that the psychiatrist’s duty extended to all foreseeable victims, not just readily identifiable potential victims. The court stated that the psychiatrist in Volk had “a duty to protect anyone who might foreseeably be endangered by the patient’s ‘dangerous propensities.’”

The lone dissenting justice in Volk objected to the court’s broadening of the duty to warn without articulating the “precise scope of this new duty, to whom it will apply, and why we make such a change.”

Emergency department patients often make threats when influenced by drugs, alcohol, or anger. Since emergency physicians are usually meeting their patients for the first time it is very difficult to assess the seriousness of the threat and even more challenging to identify any foreseeable victims. When an emergency physician evaluates a patient he or she clearly establishes a duty to that particular patient, but at what point a duty to third parties is established is more difficult to define. In tort law the likelihood of harm is not enough; the likelihood must also be foreseeable. Foreseeability often involves considerations such as the ability to “anticipate future events or to anticipate dangerous conditions that already exist.” This foreseeability is difficult to define but often focuses on what a person should have known at the time of alleged negligence. Such determinations are fact specific and vary from case to case. The decision of whether or not something is foreseeable is left to the factfinder (jury or judge depending on the type of trial). To find a person liable for negligence in a duty-to-warn case, the factfinder will be asked to decide whether the harm that occurred was reasonably foreseeable by the person accused of negligence.^6^ What remains unclarified in case law or statutes is exactly how a physician could possibly identify and specifically warn any foreseeable victim of a mentally ill or potentially violent patient.

#### Dr. Berkeley

In the landmark Tarasoff case the court also iterated a duty to warn those with infections or other diseases. In an illustrative, classic court case a patient presented to an ED with a headache, fevers and chills, and myalgias, and was admitted. His condition deteriorated and he died four days later due to Rocky Mountain spotted fever (RMSF). Throughout the course of the patient’s treatment, his physician had never informed his wife that her husband had died from RMSF, which is transmitted through the bite of an infected tick. A week after the death of her husband, she was admitted to another hospital with similar symptoms and, despite treatment for presumed RMSF, she died three days later. Her son brought suit against the first physician for negligence in failing to warn his mother that she was at risk of exposure to RMSF. During a jury trial, a plaintiff’s expert testified that family members of patients with RMSF are at risk of contracting the disease due to the geographic clustering activity of infected ticks, and a verdict was returned against the physician defendant.

The Tennessee Supreme Court subsequently granted an appeal “to determine whether a physician has a legal duty to warn a non-patient of the risk of exposure to the source of his patient’s noncontagious disease.” In its decision, the court noted that although RMSF is not contagious “it is likely that others in the patient’s household may have come into contact with infected ticks.” The court concluded that a physician has “an affirmative duty to warn identifiable third persons in the patient’s immediate family against foreseeable risks emanating from a patient’s illness...” and held that Dr. Daniel “had a duty to warn his patient’s wife of her risk of contracting Rocky Mountain Spotted Fever...”

This case serves as a cautionary tale of the duty to warn third parties of the risks relating to infectious diseases. The court’s decision is alarming due to the fact that RMSF is not transmissible between humans; thus, the infected patient’s wife was not in danger of contracting the disease from her husband. However, the court held that the physician had a legal duty to warn the patient’s wife of her “foreseeable risk” of potential exposure to infected ticks and contracting RMSF. It must be recognized that this duty may place a significant burden on a provider. Although this case did not happen to involve an emergency physician, the key lesson to take away is that physicians may be liable if they fail to warn identifiable members of a patient’s immediate family if they are foreseeably at risk of exposure to the patient’s disease. From the risk management perspective, such notification should be documented in the medical record.^7^

#### Dr. Moore

The Tarasoff case also mandated a duty to warn when medications and their side effects may lead to harm to others. This duty is defined further in the following two legal cases.

In the first case, a 12-year-old boy was diagnosed with attention deficit hyperactivity disorder ADHD by his physician and it was decided to begin desipramine (Norpramin). The physician testified that she showed the patient’s mother an entry for tricyclic antidepressants in the *Physician’s Desk Reference*. The entry described common side effects associated with the group of antidepressants, such as dry eyes and mouth and increased pulse rate. The physician also explained that the child should be watched closely for rapid heartbeat. Two years later, after multiple medical visits to a variety of settings, for multiple complaints, the child died from hypereosinophilic syndrome, which is a rare but known complication of desipramine. The parents brought suit against Walmart alleging that it was negligent in the sale of desipramine “by failing to properly warn intended users of the hazards and harms associated with the use of the product.” The court ruled that the pharmacist had no duty to warn the patient of side effects. The physician was held liable for $1.012 million.^6^

Thus, a pharmacist is not held to have a duty to warn a patient of side effects; this is considered the physician’s responsibility. Multiple state courts have reached the same conclusion. Courts feel that “to impose a duty to warn on the pharmacist would be to place the pharmacist in the middle of the doctor-patient relationship, without the physician’s knowledge of the patient.”^6^

The emergency physician erroneously may think that the pharmacist will tell the patient what side effects to watch for, and put labels on the bottles. Although this may happen, the courts do not feel this is the pharmacist’s duty or obligation.^8^

In a second case, a 52-year-old woman came to the ED with chronic migraines and was given nalbuphine (Nubain) and promethazine (Phenergen) in dosages that had been administered to the same patient 200 times before in the ED. No warning was given to the patient. One hour after discharge, she was involved in a single-car motor vehicle accident that left her a quadriplegic. The patient recovered $1.3 million, despite the fact that she appeared alert at discharge.^9^

Not all states recognize the concept of the duty to warn or have variations of the doctrine. It behooves providers to either know their state’s law or more simply warn in every situation and not fret over their particular state’s statute. A list of state laws regarding the duty-to-warn mandate follows below.^10^

- States that mandate duty to warn: Arizona, California, Colorado, Connecticut, Delaware, Idaho, Illinois, Indiana, Iowa, Kentucky, Louisiana, Maryland, Massachusetts, Michigan, Minnesota, Missouri, Montana, Nebraska, New Hampshire, New Jersey, New York, Ohio, Pennsylvania, Tennessee, Utah, Vermont, Virginia, Washington, West Virginia, Wisconsin.
- States that are “permissive” (may report, not required): Alaska, Arkansas, District of Columbia, Florida, Hawaii, Kansas, Mississippi, New Mexico, Oklahoma, Oregon, Rhode Island, South Carolina, South Dakota, Texas, Wyoming.
- No duty to warn: Maine, Nevada, North Carolina, North Dakota.
- No state position: Georgia.

## CONCLUSION

We have presented medical-legal cases that define a physician’s duty to warn and include caveats on medical practice within the scope of the law. Some physicians may not recognize that these legal and liability requirements extend not only to physical danger but also to infectious diseases, medical illness, and drug effects.

### Take-home Points

1. “Duty to warn” encompasses a broad area of responsibility for emergency physicians including not only physical harm but also harm from medications and infectious diseases.
2. The key legal concept is if the injured party is “foreseeable.” Foreseeability is subject to wide and uncertain interpretation by both juries and judges.
3. With regard to duty to warn on medications, the physician is obligated to warn of risks related to the drug; the pharmacist is tasked with safely dispensing the medication.
4. Not all state laws acknowledge the duty to warn, but it behooves physicians to comply and have less concern about possible liability.^1^
